# Spatial confinement alters morphology, spreading dynamics, and mechanics of adherent platelets

**DOI:** 10.1016/j.bpr.2025.100222

**Published:** 2025-07-24

**Authors:** Johanna G. Rodríguez, Jan Seifert, Vincent Gidlund, Carmela Rianna, Tilman E. Schäffer

**Affiliations:** 1 Institute of Applied Physics, University of Tübingen, Tübingen, Germany

## Abstract

Platelets are small blood cells involved in hemostasis and wound healing. After activation, platelets interact with their surrounding environment and respond to biochemical and mechanical stimuli by mechanosensitive and haptotactic mechanisms. We used microcontact printing (μCP) to mimic the physiological conditions and limited space in small blood vessels in vitro. With μCP, we created 4-μm-wide fibrinogen lines to provide a spatially confined spreading space for platelets. We then let platelets adhere and spread on these lines while imaging them with optical microscopy and scanning ion conductance microscopy (SICM)*.* Confined platelets showed significantly altered morphology, spreading dynamics, and mechanics compared with control platelets. Altered mechanical properties of confined platelets revealed reorganization of the actin cytoskeleton and the formation of regions of increased elastic modulus at the edges of the fibrinogen lines. Our results indicate that spatial confinement affects platelet mechanics and morphology on a subcellular level.

## Why it matters

Platelets are small cells in the blood that help to stop bleeding by forming clots. They operate in very tight spaces in the body, such as small blood vessels, where their movement and shape are physically limited. We wanted to understand how this kind of spatial confinement affects platelet function. Using precise surface patterns made from a clotting protein called fibrinogen, we recreated this narrow environment and observed how the platelets responded. We found that confinement altered their shape, the way they spread, and their mechanical response to the environment. Improving our understanding of how platelets behave in these constrained conditions could enhance our knowledge of clotting processes in health and disease.

## Introduction

Platelets play a vital role in the context of vascular injury due to their involvement in clot formation and hemostasis (1). Platelets are immediately recruited in response to endothelial injury or inflammatory signals (2). Upon activation by platelet agonists such as thrombin, platelets adhere and spread to an underlying surface by forming filopodia and lamellipodia. This leads to multicellular aggregation and thrombus formation (3). Altered platelet activation is associated with pathological cardiovascular conditions such as bleeding, thrombosis, stroke, and myocardial infarction (3,4).

Platelet mechanics and its dependence on the extracellular environment are important for the understanding of platelet function (5). The platelet cytoskeleton generates contractile forces and profoundly contributes to mechanotransduction, haptotaxis, and motility (2,6,7,8). Consequently, the mechanical, biochemical, and topographical properties of the substrate can influence platelet activation, adhesion, and migration (9,10,11,12).

Microcontact printing (μCP) enables the investigation of cell and platelet behavior in a spatial confining environment. Creating line patterns that ideally resemble the dimensions of blood capillaries using μCP allows platelet adhesion and spreading to be mimicked under conditions close to those in vivo (13). This is particularly valuable for studying platelet behavior, because platelet experiments are usually carried out on flat, unpatterned surfaces. Further applications of μCP include studying the migration of tumor cells, mesenchymal stem cells (14,15), and metastatic breast cancer cells to explore the effect of spatial confinement on cellular behavior (16). μCP is thus a powerful tool that facilitates the development of versatile, biologically relevant patterns (17).

In this study, we investigated how platelet morphology and mechanics are affected by spatial confinement using optical microscopy and scanning ion conductance microscopy (SICM) at the subcellular level. We used μCP to fabricate 4-μm-wide fibrinogen lines to provide a model system for platelet confinement in narrow blood capillaries (18).

We found that platelets were strongly affected by spatial confinement, exhibiting an elongated shape with an increased aspect ratio. During spreading, platelets slowly adapted to the confining environment, leading to an increased spreading time. Moreover, confinement caused a rearrangement of the cytoskeleton and the formation of actin-rich, stiff regions near the edges of the fibrinogen lines. Our results indicate that spatial confinement affects platelet morphology and biomechanics on a subcellular level.

## Materials and methods

### Silicon master preparation

**Figure 1:**
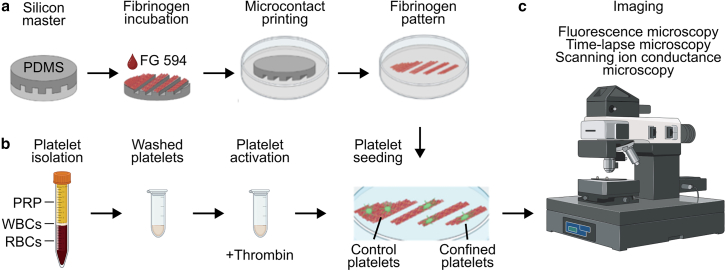
Experimental procedure. (*a*) Fabrication of patterned fibrinogen substrates by microcontact printing (μCP). A PDMS stamp, made from a silicon master and coated with fluorescent fibrinogen (FG 594), was used to imprint a line pattern onto a glass surface. (*b*) Platelets were isolated from whole blood, activated, and seeded onto a patterned fibrinogen substrate. (*c*) Experiments were conducted using optical fluorescence and time-lapse microscopy as well as SICM.

A silicon wafer was spin coated with a negative epoxy photoresist (SU8-3005) for 30 s at 3000 rpm and then transferred onto a preheated (60°C) hot plate. After 60 s, the temperature of the hot plate was quickly increased to 95°C and the wafer was prebaked for 180 s. Using a maskless lithography setup (μMLA, Heidelberg Instruments Mikrotechnik, Heidelberg, Germany), the coated wafer was exposed to UV light (365 nm, 200 mJ/cm^2^) in regions specified by a pattern designed in the GDS2 layout editor KLayout (https://github.com/KLayout/klayout). Afterward, the wafer was placed on a preheated (60°C) hot plate for 60 s, before quickly increasing the temperature of the hot plate to 95°C and post baking the wafer for 120 s. Afterward, the wafer was cooled down to room temperature, placed in a glass dish containing 1-methoxy-2-propanol acetate (PGMEA), and gently vortexed for 60 s. Then the wafer was rinsed with PGMEA, transferred to a new glass dish containing PGMEA, and again gently vortexed for 60 s. Then the wafer was rinsed again with fresh PGMEA and immediately dried under nitrogen flow. For an anti-adhesive coating, 1–2 drops of trichloro(1*H*,1*H*,2*H*,2*H*-perfluorooctyl)silane were pipetted into a heated (125°C) chamber containing the developed wafer under nitrogen atmosphere. After 30 min, the chamber was cooled down to room temperature. The wafer pattern then consisted of parallel, rectangular channels with a width of 5 μm, a depth of 5 μm, and a periodicity of 15 μm. Additionally, the same wafer pattern also provided a flat area without channels. PDMS Sylgard 184 (Avantor, Radnor, PA) was prepared with a mixing ratio of 10:1 for monomer and cross-linker. The mixture was first centrifuged at 1200 × *g* for 3 min to remove large air bubbles and then poured over the silicon wafer (Fig. 1 *a*, *left*). The sample was then placed in a desiccator for 30 min to remove remaining air bubbles and then cured for 2 h at 80°C. Afterward, the PDMS layer was removed from the silicon master and cut into smaller parts providing stamps for μCP. Additional stamps with smaller and larger line widths (used in Fig. S3) were fabricated in the same way.

### Preparation of patterned fibrinogen substrates with μCP

**Figure 2:**
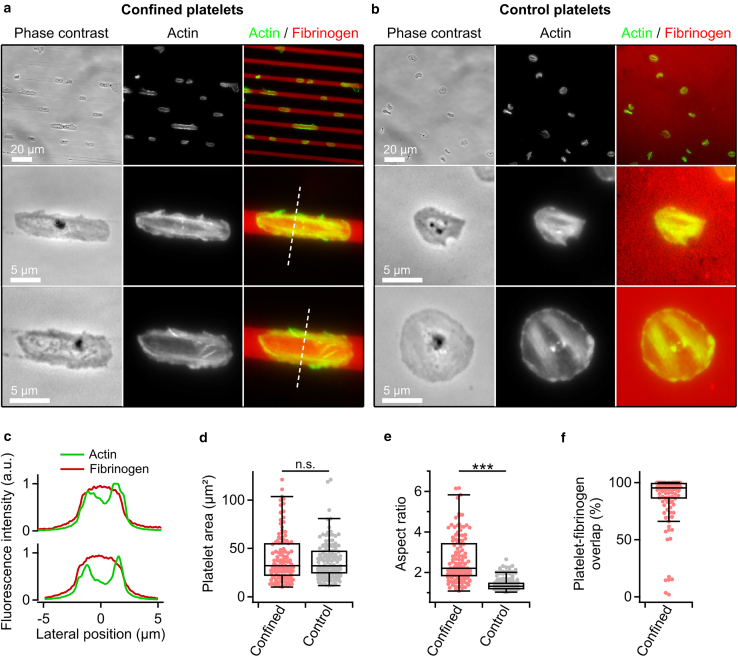
Spatial confinement of platelets. Optical phase contrast and fluorescence microscopy images (*green*, F-actin; *red*, fibrinogen) of platelets (*a*) on a line-patterned fibrinogen substrate (confined platelets) and (*b*) on a fully covered fibrinogen substrate (control platelets). (*c*) Line profiles of the fluorescence intensity along the dashed lines in (*a*). (*d*) Platelet area and (*e*) aspect ratio of platelets in confined and control conditions. (*f*) Platelet-fibrinogen overlap of confined platelets. Number of platelets: *n* = 120 (confined) and *n* = 137 (control) from 3 different donors. ∗∗∗*p* < 0.001, statistically significant difference; n.s., no significant difference (Dunn’s test). Data are presented as median ± quartiles. Whiskers indicate data points within the inner fences.

PDMS stamps were washed with 70% ethanol, dried with pressurized air, and cleaned with an oxygen plasma cleaner (Zepto-QRS 200, Diener electronic, Ebhausen, Germany) for 30 s. Afterward, fluorescent fibrinogen (66 μg/mL, Alexa Fluor 594 conjugate, F1391, ThermoFisher, Waltham, Massachusetts, USA) was used to coat the PDMS stamps for 30 min at 37°C (Fig. 1 *a*). Afterward, the stamps were washed with sterile water and dried in a flow of nitrogen gas. Cell culture glass-bottom dishes (81218, ibidi, Grafeling, Germany) were plasma-cleaned with the same procedure as described above. Then, the fibrinogen-coated PDMS stamps were placed in the centers of the cell culture dishes with the coated side facing downward. The stamps were pressed down slightly with the fingertip and the dishes were incubated for 15 min at 37°C. This created both a fully covered fibrinogen region and a line-patterned fibrinogen region (Fig. 1 *a*). The PDMS stamps were then removed, and the cell culture dishes were incubated with PEG-grafted poly-L-lysine (PLL-g-PEG, 10 μg/mL in 10 mM HEPES; SuSoS AG, Dübendorf, Switzerland) for 1 h to passivate the fibrinogen-free regions of the cell culture dishes to reduce platelet adhesion in these regions. Excess of PLL-g-PEG was removed by washing three times with phosphate-buffered saline (PBS). The line width of the resulting fluorescent pattern was about 4 μm (Fig. 2 *c*), slightly smaller than the width of the structures in the silicon master due to rounding of the PDMS edges (Fig. S1).

### Isolation of human platelets

All procedures were approved by the institutional ethics committee (273/2018BO2) and are in accordance with the Declaration of Helsinki. Informed consent from all donors was obtained before blood withdrawal. Fresh venous blood was collected from the antecubital vein of healthy volunteers in monovettes containing acid-citrate-dextrose (ACD) at 4:1 ratio (blood/ACD). Platelet-rich plasma was obtained from whole blood by centrifugation at 200 × *g* for 20 min at room temperature (Fig. 1 *b*). Tyrode-HEPES buffer (136.8 mM NaCl, 2.6 mM KCl, 8.4 mM NaHCO_3_, 5.5 mM D-glucose, 6 mM HEPES [pH 6.5]), was added to the platelet-rich plasma at a ratio of 3:1 and centrifuged again at 920 × *g* for 10 min at room temperature without brake. Finally, the supernatant was discarded, and the platelet pellet was carefully resuspended in 1 mL of Tyrode-HEPES (pH 7.4).

### Platelet activation and spatial confinement

Washed platelets were activated with thrombin (0.1 U/mL, T6884, Sigma-Aldrich, St. Louis, MO) for 2 min at 37°C. The platelets were then added to the fibrinogen-coated cell culture dishes containing Tyrode-HEPES buffer, pH 7.4. Samples were now either used for experiments (Fig. 1 *c*) or further incubated for 25 min at 37°C to allow platelet adhesion and spreading. Finally, nonadherent platelets were removed by washing with Tyrode-HEPES buffer.

### F-actin immunostaining

Platelets were fixed in PBS containing 2% formaldehyde (F1635, Sigma-Aldrich) for 10 min. Afterward, the samples were washed three times with PBS and incubated with 0.1% Triton X-100 (93443, Sigma-Aldrich) in PBS for 10 min. The samples were again washed three times with PBS and F-actin was stained with phalloidin (ActinGreen 488, diluted 1:2000 in PBS, R37110, Thermo Fisher) for 20 min at 37°C.

### Epifluorescence imaging and deep learning platelet morphometry

Phase contrast time-lapse image sequences of live platelets and phase contrast and epifluorescence images of F-actin-stained platelets and fibrinogen substrates were recorded using an inverted optical microscope (Ti-E, Nikon, Tokyo, Japan) equipped with a motorized XY stage, a 100× oil immersion objective, and a monochrome digital camera (DS-Qi2, Nikon). Phase contrast and epifluorescence images were recorded with exposure times of 50 and 300 ms, respectively. For time-lapse sequences, images were recorded every 15 s from different regions of the sample. Platelet shape parameters were extracted from phase contrast images by deep learning platelet morphometry (19). Spreading platelets were tracked using a nearest neighbor algorithm in time-lapse sequences (19). Platelets were defined as “spreading” if their initial area increased by at least a factor of 2 during the observation time. Nonspreading platelets were excluded from the analysis. For the calculation of the spreading time of single platelets, the initial and final areas were defined as 0 and 100%, respectively. The spreading time was then defined as the time it takes for the area to increase from 25 to 75%. The alignment angle was calculated with respect to the direction of the fibrinogen pattern for confined and control platelets. The major and minor axes of platelets were obtained by fitting an ellipse of the same area as the platelet to its shape. The early growth rates of the major and minor axes were calculated as the average slope of these quantities versus time in the first 5 min of spreading. Calculations were done using Igor Pro 9 (WaveMetrics, Lake Oswego, OR).

### SICM

SICM imaging was performed using a self-built SICM setup (20). Nanopipettes were fabricated from borosilicate glass capillaries (1B100F-4, World Precision Instruments, Sarasota, Florida, USA) using a CO_2_ laser-based nanopipette puller (P2000, Sutter Instruments, Novato, California, USA) and had an inner tip diameter of typically 180 nm and an inner opening angle of typically 3°. A voltage of 200 mV was applied between the electrodes and the ion current was measured with 3 kHz bandwidth. For mechanical measurements, a constant pressure of 10 kPa was applied to the upper end of the electrolyte-filled nanopipette. The images were acquired with a *z*-approach speed of 20 μm/s and an ion current trigger of 98% of the saturation current in a raster pattern with a typical pixel size of 0.4 μm, resulting in a typical image duration of 2 min. At each pixel, the ion current was recorded as a function of the *z*-position of the nanopipette (*I*-*z* curves). An image of sample topography was obtained as the *z*-position at the time point of the trigger for all pixels. An image of sample “stiffness” in terms of the elastic (Young’s) modulus was obtained from the slope in an *I-z* curve between 99 and 98% of the saturation current for all pixels (21,22). The topography images were then corrected for tilt and the platelets were identified via a height criterion (>50 nm above the substrate). By applying a correction factor to the elastic modulus, dependent on the platelet height, the influence of the underlying stiff substrate on thin sample regions was eliminated (Fig. S2) (23). The elastic modulus of a single platelet was calculated by taking the median value of the elastic modulus within the platelet area in the image. The platelet height was calculated from the topography images as the 99th percentile of the height values and the platelet volume was calculated as the mean height of the platelet multiplied with the platelet area. Additionally, the height skewness was calculated, which indicates the ratio of flat to elevated regions on the platelet surface (a height skewness of 0 indicates an equal amount of flat and elevated regions, and a positive height skewness indicates a predominant amount of flat regions).

### Statistics

Data were analyzed and processed in Igor Pro 9. Data in boxplots are presented as median ± quartiles. Whiskers indicate data points within the inner fences (upper quartile +1.5 × IQR and lower quartile –1.5 × IQR, with interquartile range IQR = upper quartile – lower quartile). The results were tested using Dunn’s test for nonparametric multiple comparisons, unless stated otherwise. Results were considered significantly different for *p* values <0.05.

## Results

### Platelet shape and spreading dynamics are altered in confinement

We examined the morphology of platelets spread on either the line-patterned fibrinogen region (confined platelets) or the fully covered fibrinogen region (control platelets). Confined platelets had an elongated morphology aligned with the fibrinogen pattern (Fig. 2 *a*). The cytoskeleton showed actin-rich regions located mainly along the edges of the fibrinogen lines (Fig. 2 *c*). Control platelets showed a roundish, spread-out morphology (Fig. 2 *b*). Here, the actin-rich regions extended across the platelet body. Confined platelets had a similar platelet area of around 30 μm^2^ compared with control platelets (Fig. 2 *d*), but a significantly (*p* < 1.06 × 10^–15^), about 2.5-fold increased aspect ratio due to the elongated shape (Fig. 2 *e*). Confined platelets showed an overlap with the fibrinogen-covered regions of > 96% (Fig. 2 *f*). Compared with the line width used in this study, narrower lines caused platelets to extend into uncoated regions, resulting in a decreased fibrinogen overlap (Fig. S3 *a*). In contrast, for a larger line width, the overlap with the fibrinogen-covered regions remained similar to that observed for the 4-μm lines, but platelets were only partially confined (Fig. S3 *b*). This confirms the findings of previous studies (13,24).

**Figure 3:**
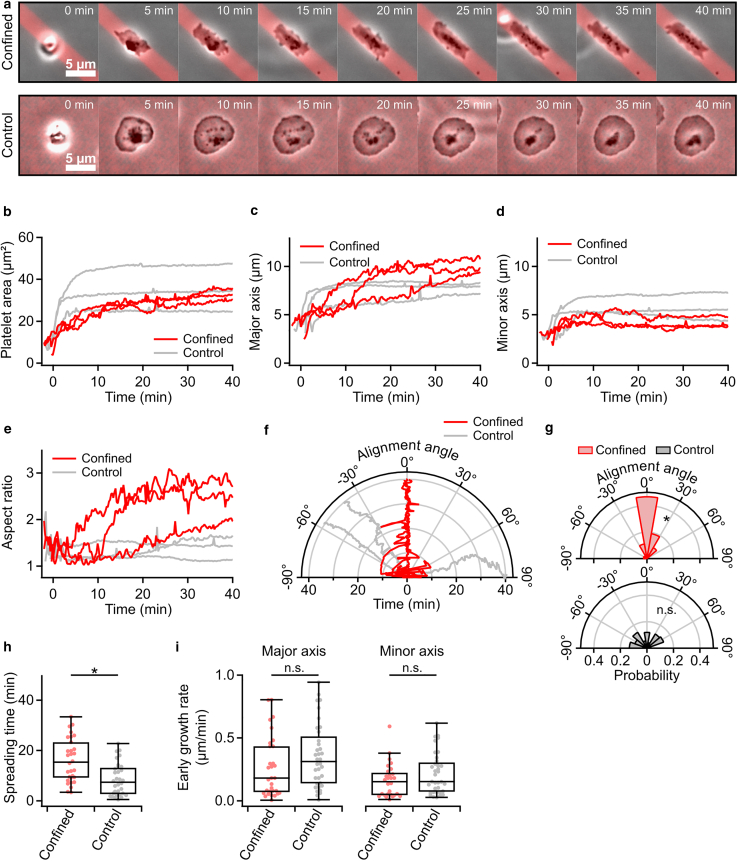
Spatial confinement influences platelet spreading dynamics. (*a*) Phase contrast image sequence of platelets during spreading in confined and control conditions. Fibrinogen-coated areas are shown as a red overlay. For the full image sequence see Video S1. (*b*) Platelet area, (*c*) major axis length, (*d*) minor axis length, (*e*) aspect ratio, and (*f*) alignment angle as a function of time during spreading. The spreading process begins at time *t* = 0 s, at which point the platelet area has increased by 25% compared with the initial area. (*g*) Alignment angle at *t* = 40 min. (*h*) Platelet spreading time, (*i*) early major and minor axis growth rates of spreading platelets. Number of platelets: *n* = 30 (confined) and *n* = 38 (control) from 3 different donors. ∗*p* < 0.05, ∗∗*p* < 0.01, ∗∗∗*p* < 0.001, statistically significant difference. n.s., no significant difference. Data in (*h*) and (*i*) were tested for significance using Dunn’s test. Data in (*g*) were tested for uniformity using Rayleigh’s test. Data are presented as median ± quartiles. Whiskers indicate data points within the inner fences.

We then investigated the effect of spatial confinement on the spreading dynamics of platelets using time-lapse phase contrast microscopy. Both confined and control platelets adhered and adopted a spread-out morphology within 40 min (Fig. 3 *a*; Video S1). While control platelets showed a rapidly increasing platelet area during the first 5 min, confined platelets showed a more gradual increase in platelet area (Figs. 3 *b* and S4). A refined analysis of the spreading process showed that the minor axis length increased for 5 min up to 4 μm, congruent with the fibrinogen line width, while the major axis length continued to increase continuously, to a final value larger than for the control (Fig. 3, *c* and *d*). Consequently, the aspect ratio of confined platelets increased after 5 min, while the control remained on a constant, low level (Fig. 3 *e*). Additionally, confined platelets aligned with the fibrinogen pattern within an angular range of ±15° (*p* = 4.6 × 10^–10^), whereas control platelets had a random orientation (Fig. 3, *f* and *g*). The spreading time of confined platelets (15 min) was significantly (*p* = 0.017) increased compared with control platelets (8.7 min) (Fig. 3 *h*). Interestingly, the early growth rates along the major and minor axes during the first 5 min of spreading were similar for confined and control platelets (Fig. 3 *i*). In agreement with the analysis of fully spread platelets shown in Fig. 2, the initial (at adhesion) and final (*t* = 40 min) platelet areas of confined and control platelets were similar (Fig. S5, *a* and *b*). The final aspect ratio at *t* = 40 min of confined platelets was significantly (*p* = 1.1 × 10^–4^) increased (Fig. S5 *c*).

Video S1. Phase contrast image sequence of platelets during spreading in confined and control conditions from Fig. 3 *a* Images of the fibrinogen-coated areas were recorded by fluorescence microscopy before the image sequence and are shown as a red overlay. Images were recorded with a 100× oil immersion objective every 15 s.

### Spatial confinement affects the mechanical properties of platelets

**Figure 4:**
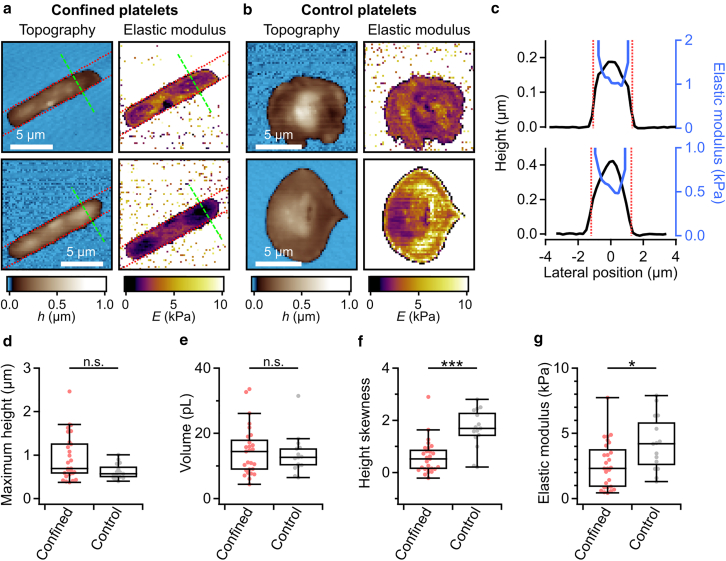
Spatial confinement alters platelet morphology and mechanics. Topography (*left*) and elastic modulus (*right*) of (*a*) confined and (*b*) control platelets by SICM. The dotted red lines indicate the edges of the imprinted fibrinogen line. The green dashed line indicates the position of the line profiles in (*c*). (*c*) Line profiles of platelet topography and elastic modulus along the green dashed lines in (*a*). (*d–f*) Morphological parameters calculated from topography images: Maximum height, volume, and height skewness of confined and control platelets. (*g*) Elastic modulus of confined and control platelets calculated from elastic modulus images. Number of platelets: *n* = 27 (confined) and *n* = 15 (control) from 3 different donors. ∗*p* < 0.05, ∗∗∗*p* < 0.001, statistically significant difference; n.s., no significant difference. Data were tested for significance using Dunn’s test. Data are presented as median ± quartiles. Whiskers indicate data points within the inner fences.

We investigated the three-dimensional morphology and the mechanics of platelets in confinement by SICM. The pressurized nanopipette allows a contact-free measurement of mechanical sample properties by gently deforming the sample surface during imaging. We recorded maps of topography and local elastic modulus of platelets (Fig. 4 *a*). Confined platelets had an elongated morphology without a clearly distinguishable lamellipodium and platelet body (Fig. 4 *a*, *left*). Accordingly, elastic modulus maps of confined platelets showed local variations that could not be clearly correlated with specific parts of the platelet (Fig. 4 *a*, *right*). In contrast, control platelets had a roundish morphology with a flat lamellipodium and higher regions at the centrally located platelet body (Fig. 4 *b*, *left*). The elastic modulus maps of control platelets show softer regions at the platelet body and stiffer regions at the lamellipodium (Fig. 4 *b*, *right*). Interestingly, confined platelets showed an increased elastic modulus toward the edges of the imprinted fibrinogen line (Fig. 4, *a* and *c*, *red dotted lines*), coinciding with the actin-rich regions (Fig. 2 *c*).

The maximum height of confined platelets was not significantly different from that of control platelets (*p* = 0.07) (Fig. 4 *d*). Likewise, confined and control platelets had a similar (*p* = 0.72) average platelet volume of about 12 and 14 fL, respectively (Fig. 4 *e*). For confined platelets, the height skewness was significantly (*p* = 9.7 × 10^-5^) reduced and close to 0 (Fig. 4 *f*), indicating a less pronounced formation of flat lamellipodia due to the limited space. On average, the elastic modulus of confined platelets (2.3 kPa) was significantly (*p* = 0.02) decreased compared with control platelets (4.2 kPa) (Fig. 4 *g*).

## Discussion

We investigated different aspects of human platelet behavior in a spatially confining environment. Microcontact-printed fibrinogen lines with a line width similar to the diameter of blood capillaries provided a substrate for platelets (Fig. 2). Using optical microscopy and SICM, we investigated alterations in morphology, spreading dynamics, and mechanics of confined platelets. Platelets adhered to and spread on the fibrinogen pattern when activated by thrombin (6,25,26) or in the presence of Ca^2+^ and Mg^2+^ (Fig. S6). Confined platelets had an elongated morphology that overlapped with the fibrinogen lines (Fig. 2), and the aspect ratio was increased to about 2. This is a similar value to that found in a previous study (13) for a similar width of fibrinogen lines as in our study. Additionally, confined platelets showed altered spreading dynamics with an increased spreading time (Fig. 3) and altered mechanical properties (Fig. 4). Previous studies have shown that microtopography and spatial confinement influence platelet adhesion and spreading behavior (11,13,27,28). However, the dynamic process of platelet spreading in confinement has not yet been investigated in detail. Unconfined platelets typically show a rapid early area increase (29) accompanied by the formation of actin stress fibers (30). We found that the spreading area of confined platelets increased slower than that of control platelets. However, the early growth rate along the platelets’ major axis, which aligned with the fibrinogen lines, was similar to that of unconfined platelets. This suggests that only subcellular regions in direct proximity to the confining boundaries are affected by the confinement, rather than the whole cell. In addition to exhibiting altered spreading dynamics, confined platelets were softer than control platelets. Similar results are known from nucleate cells, which become softer in spatial confinement (31,32,33) and exhibit a reduced F-actin content in the cell interior (34). Further, confined platelets showed an increased elastic modulus at the cell borders near the edges of the fibrinogen lines. An increased actin content was found in these regions, which is consistent with the observations made by Kita et al. (13) and for nucleate cells at cellular sites near the boundary of a confining environment (35).

The depth sensitivity for mechanical measurements with SICM is in the order of the pipette diameter (23), which is similar to the height in the flat regions of the platelet lamellipodium (Fig. 4, *a* and *b*). For this reason, a correction method that compensates for the influence of the underlying stiff substrate was applied (Fig. S2) (23). Nevertheless, firm cytoskeletal structures such as stress fibers near the bottom surface may have a greater influence on the measured elastic modulus in flat cell regions than in higher regions, where predominantly the cell cortex is measured. This is a general methodological limitation of scanning probe methods such as SICM.

In summary, we showed that confinement-induced cytoskeletal changes in platelets have a direct impact on their mechanical properties and spreading dynamics at a subcellular level. This confirms the feasibility of an in vitro μCP system for investigating platelet behavior, morphology, and biomechanics in a biomimetic environment (13,24).

## Acknowledgments

This work was funded by the 10.13039/501100001659Deutsche Forschungsgemeinschaft (DFG, German Research Foundation)—Projektnummer 335549539/GRK2381 and Projektnummer 374031971 - TRR 240. We acknowledge support by the High Performance and Cloud Computing Group at the Zentrum für Datenverarbeitung of the 10.13039/501100002345University of Tübingen, the state of Baden-Württemberg through bwHPC, and the 10.13039/501100001659DFG through grant no. INST 37/935-1 FUGG. We acknowledge support by the Open Access Publishing Fund of the 10.13039/501100002345University of Tübingen. We thank Dr. Ronny Löffler from the LISA+ facilities at the University of Tübingen for assistance in the production of the silicon master. Fig. 1 was created with BioRender.com.

## Author contributions

J.G.R., C.R., and T.E.S. designed the study. J.G.R., C.R., V.G., and J.S. performed the experiments. J.G.R., J.S., V.G., and T.E.S. analyzed the data and wrote the manuscript. J.G.R., J.S., C.R., V.G., and T.E.S. interpreted data, discussed results, and revised the manuscript. All authors have read and agreed to the final version of the manuscript.

## Declaration of interests

The authors declare no competing interests.

## Supporting material

Document S1. Figures S1–S6

Document S2. Article plus supporting material
