# Supplementary material for: Spatial confinement alters morphology, spreading dynamics, and mechanics of adherent platelets
Source: Biophys Rep (N Y). 2025 Jul 24;5(3):100222. doi: 10.1016/j.bpr.2025.100222 (PMC12355061; doi:10.1016/j.bpr.2025.100222)
Supplement: Document S1. Figures S1–S6 [file mmc1.pdf]

Biophysical Reports, Volume *5*

Supplemental information

Spatial confinement alters morphology, spreading dynamics, and mechanics of adherent platelets

Johanna G. Rodríguez, Jan Seifert, Vincent Gidlund, Carmela Rianna, and Tilman E. Schäffer

Supplemental Figures

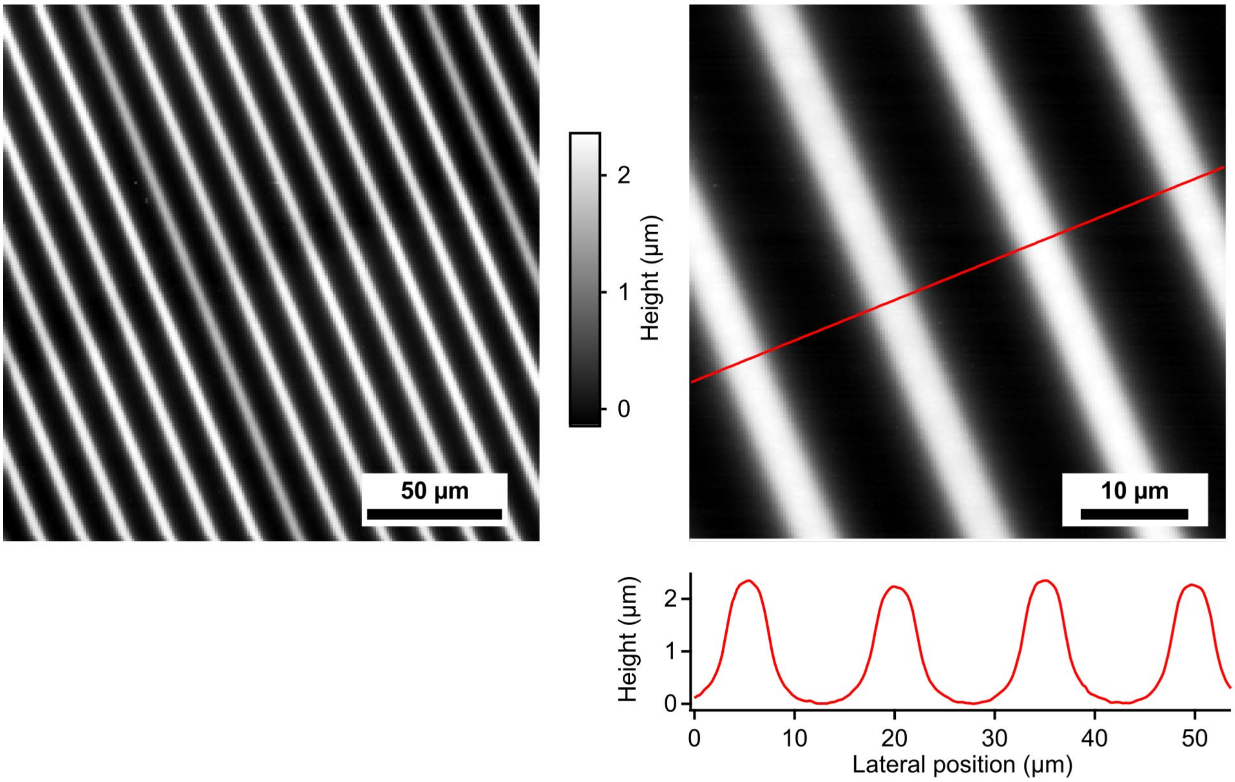

Figure S1: SICM images of an uncoated PDMS stamp for 4 µm wide lines.

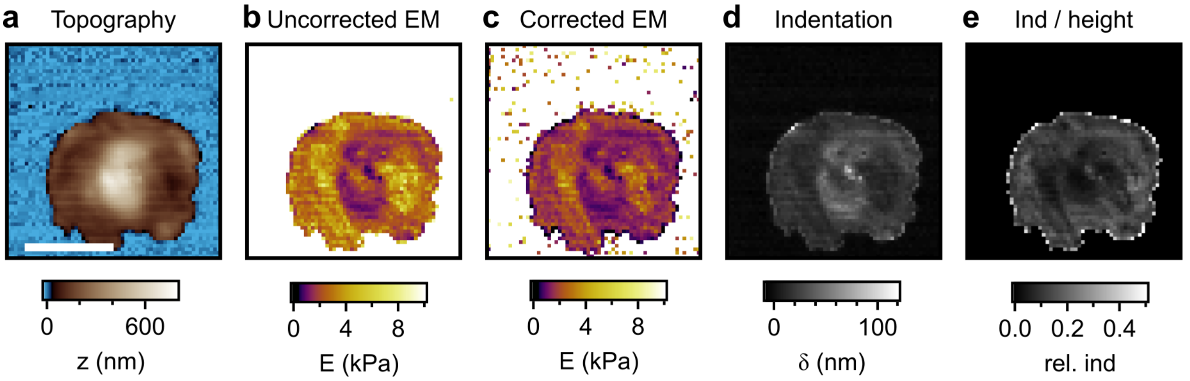

Figure S2: Mechanical measurements of platelets with SICM. (**a**) Topography and (**b**) uncorrected elastic modulus of the platelet shown in Fig. 4b, top row. (**c**) Height-corrected elastic modulus, (**d**) indentation, and (**e**) indentation normalized to the local height of the platelet.

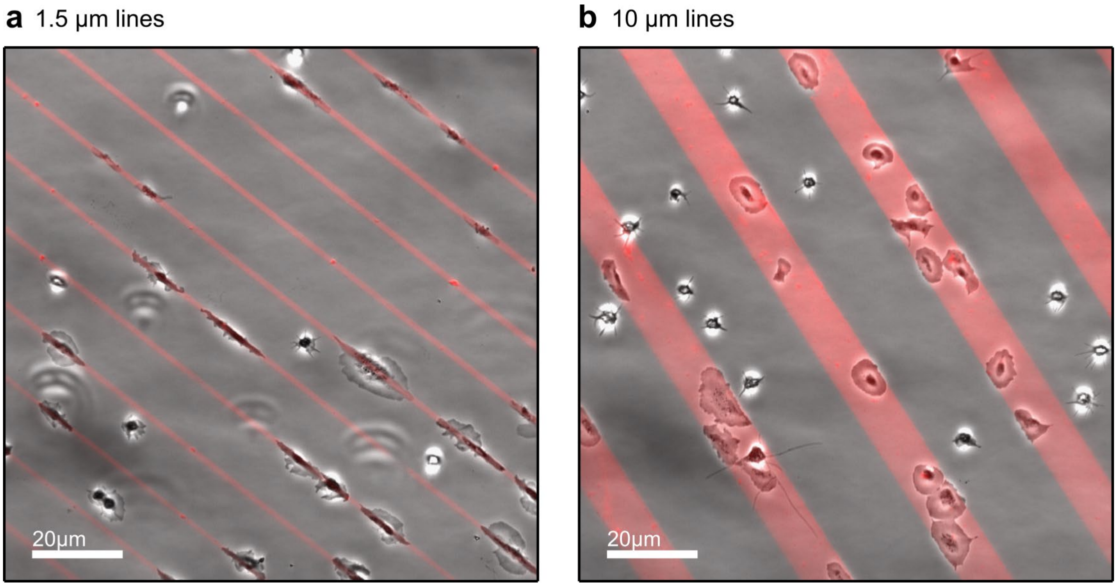

Figure S3: Platelets spread on fibrinogen lines that had a smaller (**a**, ≈1.5 µm) or a larger (**b**, ≈10 µm) line width compared to the lines used in Figs. 2-4.

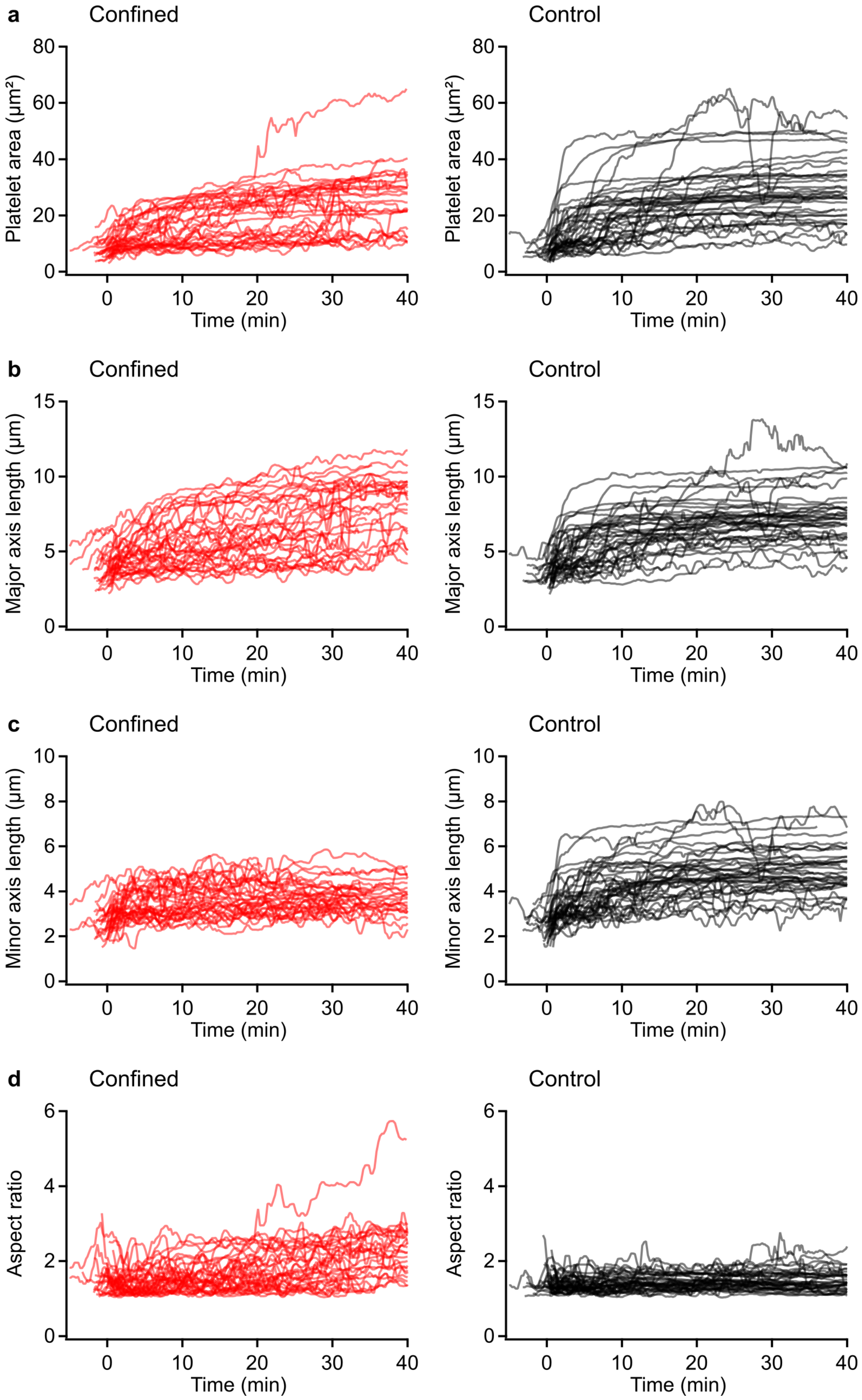

Figure S4: (**a**) Platelet area, (**b**) major axis length, (**c**) minor axis length, and (**d**) aspect ratio as a function of time during spreading for all platelets investigated in Fig. 3g-i and Suppl. Fig. S5.

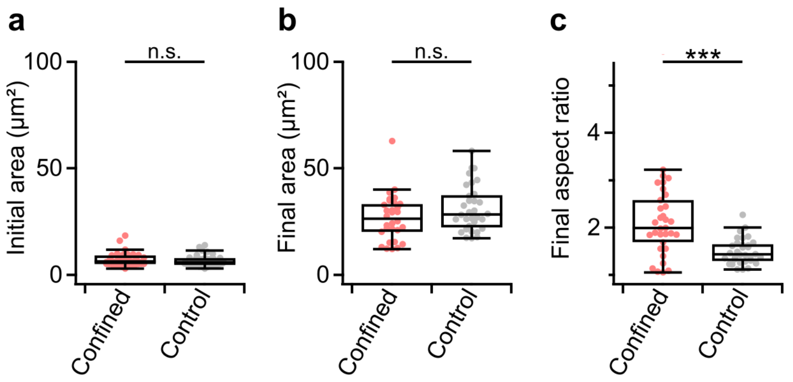

Figure S5: Additional analysis of the platelets shown in Fig. 3: (**a**) Initial platelet area after adhesion, (**b**) final platelet area at $t = 40$ min, and (**c**) final aspect ratio (at $t = 40$ min).

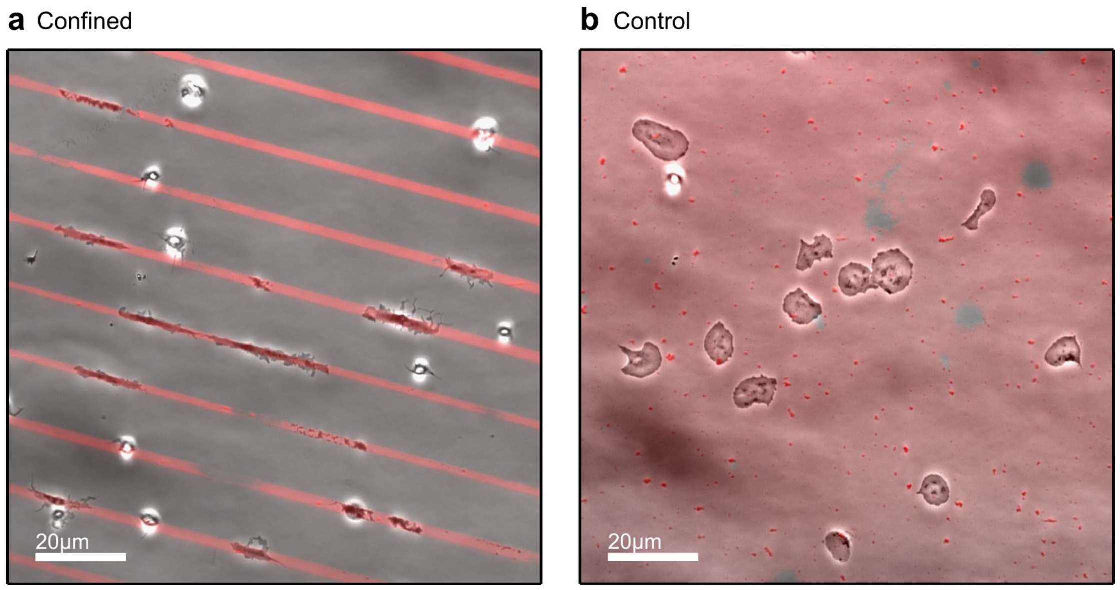

Figure S6: Platelets spread on fibrinogen without thrombin but in the presence of 2 mM Ca^{2+} and 1 mM Mg^{2+}.
